# Spatial and Spatio-Temporal Distribution of Human Respiratory Syncytial Virus, Human Parainfluenza Virus, and Human Adenoviruses Cases in Kenya 2007-2013

**DOI:** 10.24248/eahrj.v6i1.679

**Published:** 2022-07

**Authors:** Therese Umuhoza, Julius Oyugi, James D. Mancuso, Wallace D. Bulimo

**Affiliations:** a Institute of Tropical and Infectious Diseases, University of Nairobi; b Department of Preventive Medicine and Biostatistics, Uniformed Services University of the Health Sciences, Bethesda, MD, USA; c Center of Virus Research, Kenya Medical Research Institute

## Abstract

**Background:**

Human Respiratory Syncytial Virus (HRSV), Human Parainfluenza Virus (HPIV), and Human Adenovirus (HAdV) epidemics differ in geographical location, time, and virus type. Regions prone to infections can be identified using geographic information systems (GIS) and available methods for detecting spatial and time clusters. We sought to find statistically significant spatial and time clusters of HRSV, HPIV, and HAdV cases in different parts of Kenya.

**Methods:**

To analyse retrospective data, we used a geographical information system (GIS) and the spatial scan statistic. The information was gathered from surveillance sites and aggregated at the county level in order to identify purely spatial and Spatio-temporal clusters. To detect the presence of spatial autocorrelation, the local Moran's I test was used. To detect the spatial clusters of HRSV, HPIV, and HAdV cases, we performed the purely spatial scan statistic. Furthermore, space-time clusters were identified using space-time scan statistics. Both spatial and space-time analyses were based on the discrete Poisson model with a pre-specified statistical significance levelof p<0.05.

**Results:**

The findings showed that HRSV, HPIV, and HAdV cases had significant autocorrelation within the study areas. Furthermore, in the Western region of the country, the three respiratory viruses had local clusters with significant positive autocorrelation (p<0.05). Statistically, the Western region had significant spatial clusters of HRSV, HPIV, and HAdV occurrence. Furthermore, the space-time analysis revealed that the HPIV primary cluster persisted in the Western region from 2007 to 2013. However, primary clusters of HRSV and HAdV were observed in the Coastal region in 2009-11 and 2008-09, respectively.

**Conclusion:**

Human respiratory syncytial virus (HRSV), human parainfluenza virus (HPIV), and human adenovirus (HAdV) hotspots (clusters) occurred in Kenya's Western and Coastal regions from 2007 to 2013. The Western region appeared to be more prone to the occurrence of allthree respiratory viruses throughout the study period. Strategic mitigation should focus on these locations to prevent future clusters of HRSV, HPIV, and HAdV infections that could lead to epidemics.

## BACKGROUND

Acute respiratory tract infections caused by the human respiratory syncytial virus (HRSV), human parainfluenza virus (HPIV), and human adenovirus (HAdV) account for a significant portion of the global public health burden of disease^1,2^. In 2015, HRSV incidence accounted for approximately 33.1 million of the total number of acute lower respiratory tract infections (ALRTIs) in children under the age of five years worldwide.^2^ While HRSV is the most common cause of ALRTIs in children under the age of five, HPIV and HAdV also contribute to the burden of acute respiratory infections.^3,4^ These tree respiratory viruses spread efficiently among humans through direct transmission such as self-inoculation, and indirect transmission such as fomites as well as droplets or aerosols.^5^ In 2018, the global burden of HPIV–ALRTIs was estimated to be 29.5 million cases in children under the age of five.^6^ Furthermore, outbreaks caused by HAdV have raised public health concern, as emerging and re-emerging types have been linked to severe pneumonia in healthy children and adults^7,8^ Outbreaks of these respiratory viruses occur in various locations around the world, and the timing of epidemics varies depending on population and location.^9^ The variation in occurrence rates has been linked to a number of factors, including demographic, socioeconomic, and climate variables^10–12^.

Several African countries have reported data indicating a significant proportion of HRSV, HPIV, and HAdV infections. Human respiratory syncytial virus (HRSV) alone had a 14.6% prevalence in people with (acute respiratory tract infections) ARTIs across the continent, indicating that the ecosystem of this continent contributes to the epidemiology of these respiratory virus infections.^13^

Despite the lack of pooled prevalence data for HPIV and HAdVs from the continent, both viruses are typically recorded through syndromic surveillance systems such as influenza-like illness (ILI) and severe acute respiratory illness (SARI) or pneumonia.^14–16^ For the period 2000-2015, the proportion of ARTIs in children under the age of five years ranged from 4% to 35% of HRSV, 0.3% to 75.8% of HPIV, and 1% to 26% HAdV in Sub-Saharan Africa.^17^

Human respiratory syncytial virus (HRSV) epidemics have been reported in temperate regions during the coldest months of winter, whereas in tropical areas epidemics are mostly associated with rainy seasons.^18^ However, HRSV peaks have also been recorded south of the equator during a dry season.^19^ Similar to HAdV serotypes, varying seasonal peaks were observed with HPIV types (1-4), and both respiratory viruses occur throughout the year with less defined seasonality in the northern and southern hemispheres.^20,21^

Kenya, like other countries, has reported a substantial burden of disease from HRSV, HPIV, and HAdV infections. However, the identification of geographical areas prone to HRSV, HPIV, and HAdV infections, or “hotspots,” in the country is lacking. These regions can be identified using geographic information systems (GIS) and the available methods for detecting spatial and space-time clusters “hotspots”.^22^ Techniques such as scan statistics, which combines exploratory and confirmatory capabilities, enable an unambiguous statistical assessment of spatial and spatiotemporal patterns across defined regions.^23,24^

The objective of this study was to identify statistically significant spatial and time clusters of HRSV, HPIV, and HAdV infections in Kenya at the county and regional levels. The hypothesis was that HRSV, HPIV, and HAdV infections occur in clusters with both spatial and spatio-temporal characteristics.

## METHODOLOGY

### Study Areas Characteristics

The research was carried out in Kenya, which is located in Eastern Africa at a latitude of -0.0236° S and a longitude of 37.9062° E.^25^ Kenya's borders are 3,457 kilometers long, and it shares borders with South Sudan, Ethiopia, Somalia, Tanzania, Uganda, and the Indian Ocean.^26^ The equator cuts the country in half, with roughly half of Kenya located in the northern hemisphere.

**FIGURE 1:**
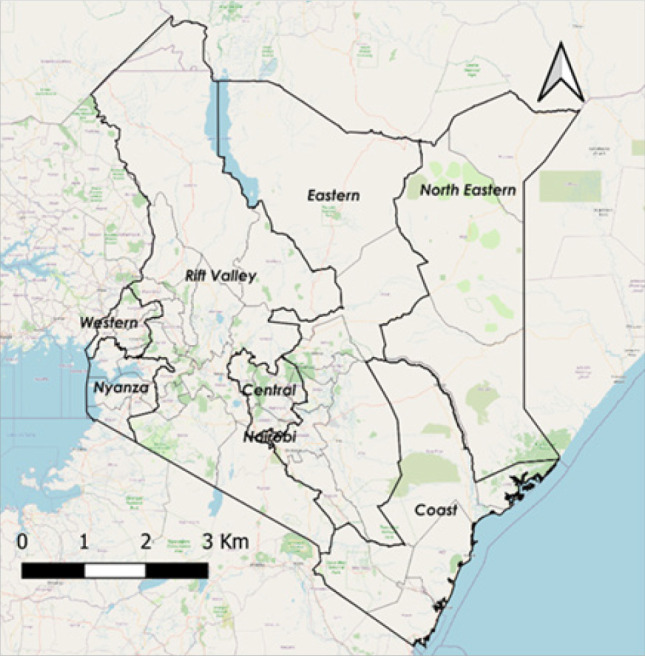
Geographical Boundaries of Kenya

Kenya had eight provinces subdivided into 158 districts prior to the adoption of the new Kenyan constitution in 2010.^27^ In 2010, the country was reorganized into 47 counties and 290 sub-counties. From 2013 onwards, this geographic boundary hierarchy was incorporated into national administration. Counties were established in accordance with the previous provinces (Figure 1), which belonged to their respective areas and populations according to the 2009 census.^28^

Kenya's coastal region (province) is divided into six counties covering an area of 79,686.1 km^2^. The average temperature in this region is 22-30°C, and the annual rainfall ranges from 20mm to 300mm.^29^ The Rift Valley region is Kenya's largest, with fourteen counties and an area of 182,505.1 Km.^2^ The climate of the Rift Valley region is characterised by arid areas or hot desert in the north, tropical savanna in the centre, and cooler temperate areas in the south. The average temperature ranges from 10°C to 28°C, with temperatures exceeding 40°C in the most arid areas. The rainfall ranges from 500mm to 3000mm, with the central region receiving the most.^30^ With eight counties, the Eastern region has the second largest area at 140,698.6 Km.^2^ The regional characteristics range from arid in the north to semi-arid in the south, with erratic temperature and rainfall variation.^29,31,32^ The North-Eastern region consists of three counties covering a total area of 127,358.5 km^2^. The climate is more akin to that of the Eastern region, with the hottest desert or arid areas in the north and semi-arid areas that cool off toward the southeast. Both regions have sparse rainfall in the north, ranging between 250mm and 500mm per year, as well as average temperatures ranging from 20°C to 40°C. The southeastern zone, on the other hand, receives the most annual precipitation, approximately 3673 mm.^29^

Kenya's central region is made up of five counties totaling 11,449.1 km^2^. The region experiences cooler temperatures ranging from 14°C to 28°C, as well as moderate rainfall ranging from 1016 mm to 2540 mm per year.^33^ Nairobi is Kenya's capital city and the county seat, with a land area of 694.9 km^2^. Nairobi's climate is more similar to that of the country's central region. The average temperature is around 19°C, and the average annual rainfall is 958 mm.^34^ Four counties account for 7,400.4 km^2^ of the area in the Western region. It has a climate with equatorial tropical features as well as some temperate savanna areas. This region has a mean annual rainfall of 2087mm, with the heaviest rain falling in April, and temperatures ranging from 14°C to 36°C.^29^ The Nyanza region borders the Western region; it has a land area of 12,477.1 km,^2^ six counties, and a similar climate as the Western region.

### Data Sources

#### Cases of human respiratory syncytial virus, human parainfluenza virus, and human adenoviruses

Human respiratory syncytial virus (HRSV), parainfluenza (HPIV), and adenoviruses (HAdV) cases were identified in this study using Kenya's influenza-like illness (ILI) syndromic surveillance system. The Kenya Medical Research Institute (KEMRI) and the US Army Medical Research Directorate - Kenya (USAMRD-K) implemented the ILI surveillance system. The case datasets were collected between 2007 and 2013. Human respiratory syncytial virus (HRSV), human parainfluenza virus (HPIV), and human adenovirus (HAdV) infections were confirmed in the laboratory using assays such as polymerase chain reaction (PCR), viral culture, and immunofluorescence. The spatial coordinates of the participants’ village/estate of origin was collected from the geocoded location via Earth Pro (7.3 Google LLC), recording latitude and longitude in degrees for each HRSV, HPIV, and HAdV case. In addition, the date of illness onset was recorded in months and years.

#### Population

The Kenya National Bureau of Statistics (KNBS) provided population data based on the 1999 and 2009 national population censuses. In the 1999 census, each district was enumerated and its population count was recorded. As a result, the following ten-year population projection from 2000 to 2010 was published as population counts per district.^35,36^ The 2009 National Population Census also recorded population counts by district. Nonetheless, population projections for each district in their respective counties have been published from 2010 to 2020.^37^ The population data used for this study in 2009 were the population counts from the 2009 census, not population projections; the projections were used for the inter-census years of 2007, 2008, 2010, 2011, 2012, and 2013. From the geocoded location, spatial coordinates including latitude and longitude centroid files of counties were created using the vector layer of Kenya administration boundaries.^38^

### Data analysis

In the first step of the analysis, the dataset was visualised during exploratory analysis to identify obvious errors and to describe the spatial distribution of HRSV, HPIV, and HAdV cases per county. The colour symbol categories were used to indicate the pattern of a large to a small number of cases per county. A higher number of cases was represented by a darker colour, while a lower number of cases was represented by a lighter colour.^22^

The second step constituted smoothing thenvisualisation. Kernel density estimation (KDE) was used for describing the spatial distribution of HRSV, HPIV, and HAdV cases in terms of the hotspots in the study area. In qGIS (V3.10.3-A Coruña), the Heatmap plugin generates a density heatmap raster from the input point of a vector layer. The density of the HRSV, HPIV, and HAdV case points is calculated based on the number of points in a location, which is the mean number of events per unit area. This allowed identifying hotspots with the use of a 10 km radius of the kernel (kernel bandwidth)^22,39^. However, from KDE it is not possible to determine the significant positive spatial autocorrelation.

In the third step, Local Moran's I test was applied to detect significant positive spatial autocorrelation from the aggregated data of HRSV, HPIV, and HAdV cases in their counties.^22^ The indicators, known as “Local Indicators of Spatial Association (LISA)” were calculated as:


(1) $$I_{i}=Z_{i}\sum_{j,j\neq i}^{n}W_{ij}Z_{J}$$

Where Z_i_ and Z_j_ are the observed values in a standardised form, and W_ij_ is a spatial weights matrix in row-standardised form. The enabled qGIS hotspots analysis plugin allowed for the execution of a local Moran's I test with a queen's cases contiguity matrix and 999 random permutations at a 5% level of significance.^40^

LISA reveals spatial clusters with values in quadrants defined as hot spots (high-high), cold spots (low-low), spatial outliers (high-low/low-high), and no significant local spatial autocorrelation.^41–44^ LISA, on the other hand, does not provide spatial cluster or hotspot characteristics.

The fourth step of the analysis consisted Kulldorff's scan statistic. This was performed to account for different aspects of spatial pattern analysis. It was based on the discrete Poisson model to identify purely spatial clusters HRSV, HPIV, and HAdV cases by counties. Besides purely spatial analysis, to determine the presence of space-time clusters of HRSV, HPIV, and HAdV cases per year over the study period, space-time scan statistic was applied. It used a Space-Time Poisson model which considered the study period of 2007-2013.

To complete the fourth step of the analysis, different datasets were prepared to fit the format used in SaTScan software (SaTScan V9.6.1). A case file containing annual HRSV, HPIV, and HAdV cases per county for a study period of 2007 to 2013 was made. A coordinate file with geographic coordinates of the centroid of each county, and a population file including the projected total population per county for each year from 2007 to 2013 were also made. These datasets were prepared in order to determine whether the incidence of HRSV, HPIV, and HAdV cases was clustering in space and time retrospectively. SaTScan uses a random process to search for and identify a significantly increased risk of HRSV, HPIV, and HAdV cases exceeding the expected number within the specified spatial window.^22,4^

The analysis was based on aggregated data per county with sites that participated in the surveillance program in Kenya from 2007 to 2013. Previously, the centroid coordinate of each county was extracted from the geocoded location of Kenya administrative boundaries vector layer with the qGIS geometry tool. By the centroids algorithm, a new point layer representing the centroids of Kenya counties was generated. The features were joint with annual HRSV, HPIV, and HAdV cases for each year to the centroid of the counties. This was completed with qGIS and later converted to SaTScan format for further cluster analysis.

To identify clusters, a cylindrical window was used with a circular geographic formbasedon each county centroid. The window moved in space and time across the study region. The spatial dimension was represented by the circular base of the cylinder with a varying radius from 0 up to the maximum value specified. The height of the cylinder constituted the temporal dimension with a time precision of 1 year. The maximum value of up to 50% of the total population at risk in space and time was considered. It was assumed that for each cylinder, HRSV, HPIV, and HAdV cases were Poisson-distributed. To assess the space-time clusters, it was assumed thatcases were randomly distributed in space and time. From the incidence of cases counted inside and outside the cylinder, the likelihood ratio was calculated for each cluster. A primary cluster was identified as the cylinder with the maximum likelihood ratio. The 999 Monte Carlo randomisations were used to obtain likelihood ratio statistics and p-values of the test. This allowed us to identify significant space-time clusters.^45^ A cluster was pre-specified to be statistically significant at the 0.05 level. Sensitivity analysis was performed using different maximum scanning window sizes to test for the robustness of the clusters. Thereafter, nvisualisation of clusters was performed in qGIS to obtain the map of the clusters' locations in study regions.

Ethical approvals for this study were obtained from Kenya Medical Research Institute (KEMRI) Scientific and Ethics Review Unit (SERU) with reference number KEMRI/SERU/CVR/003/3802 and the Walter Reed Army Institute of Research (WRAIR) with reference number WRAIR#1267G.

## RESULTS

**FIGURE 2:**
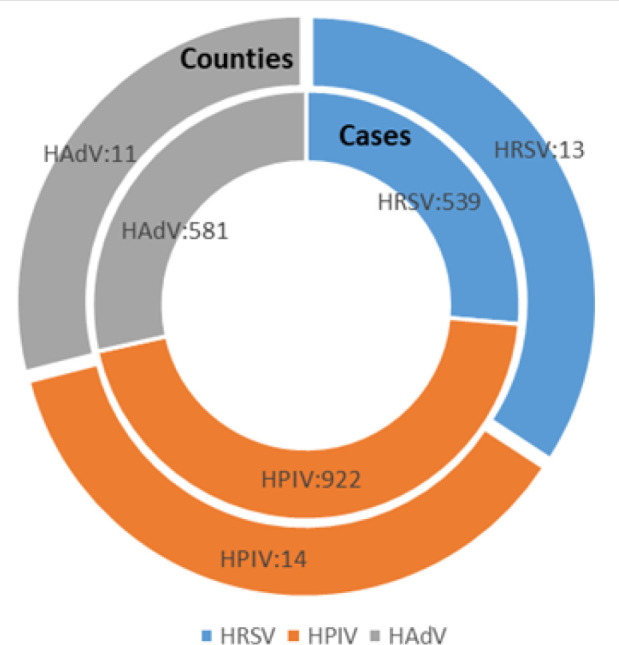
Summary Statistics of HRSV, HPIV, and HAdVs Cases and Counties in Kenya (2007-2013)

From the surveillance period of 2007-2013, HRSV was reported in 13 counties (number of cases [n]=539), and HPIV and HAdV were described in 14 (n=922) and 11 (n=581) counties respectively (Figure 2).

**FIGURE 3:**
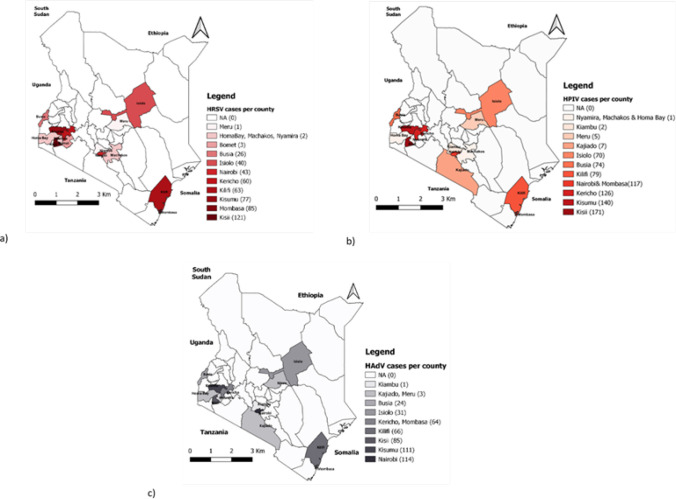
Geographical distribution of (a) Human respiratory syncytial virus (HRSV); (b) Human parainfluenza (HPIV); and (c) Human adenoviruses (HAdV) cases per counties in Kenya (2007-2013)

The geographical distribution of HRSV, HPIV, and HAdV cases varied by county (Figure 3). HRSV cases were common in Kisii county (n=121) but uncommon in Meru county. Kisii county also had the highest number of HPIV cases (n=171), while Nyamira, Machakos, and Homa Bay counties had the lowest number of cases (n=1). Nairobi county had 114HAdV cases, while Kiambu county had only one.

**NA=not applicable; No cases from these counties were captured at any surveillance site as part of the ILI program.

**FIGURE 4:**
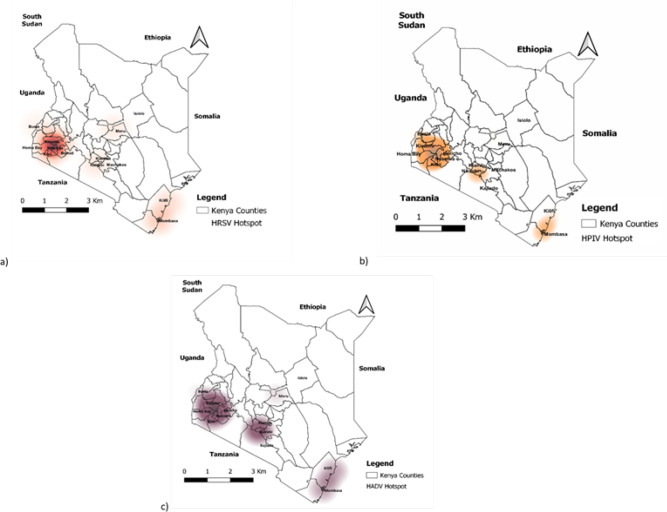
The geographical location of (a) Human respiratory syncytial virus (HRSV); (b) Human parainfluenza (HPIV); and (c) Human adenoviruses (HAdV) hotspots/clusters in Kenya (2007-2013)

The hotspot regions for HRSV, HPIV, and HAdVs cases were identified using a kernel density estimate (Figure 4). HRSV hotspots with a high density of cases were identified in the western and coastal regions.

**ILI surveillance sites were located in these counties; Kilifi, Mombasa, Nairobi, Isiolo, Kisii, Kericho, Kisumu, and Busia.

Furthermore, the coastal regions were identified as a hotspot for HPIV and HAdV cases. Nonetheless, HPIV hotspots were discovered in Kenya's central (Nairobi) and western regions from 2007 to 2013. Similarly, HAdV hotspots were observed in the central (Nairobi), and western regions.

The results of spatial autocorrelation showed significant local hotspots (clusters) for HRSV, HPIV, and HAdVs (Figire 5). The 3 respiratory viruses had local clusters with significant positive autocorrelation in the western region of the country. The central (Nairobi), and coastal regions had no significant local cluster for either HRSV, HPIV, or HAdV.

**FIGURE 5:**
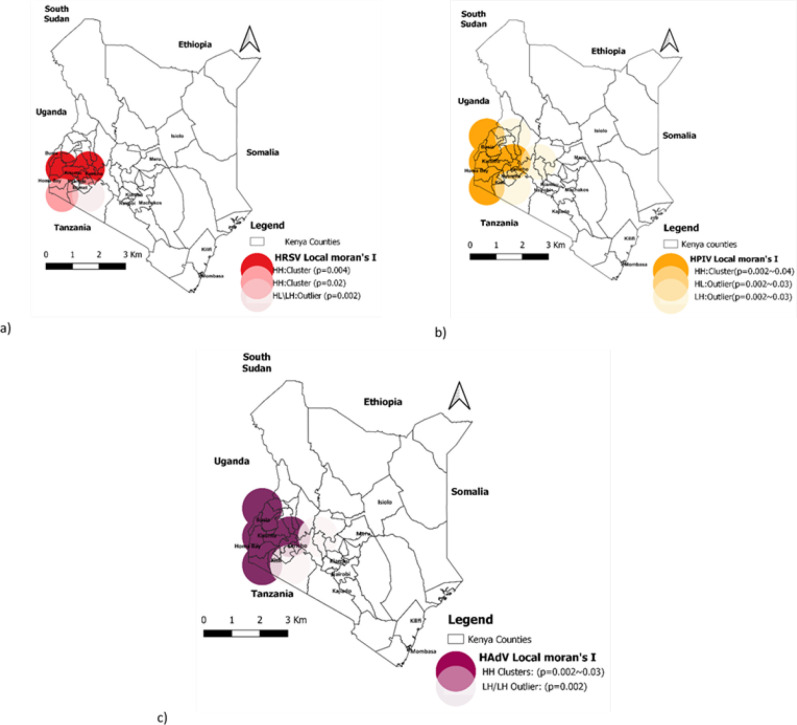
Geographical locations significantly associated with (a) Human respiratory syncytial virus (HRSV); (b) Human parainfluenza virus (HPIV); and (c) Human adenoviruses (HAdV) hotspots/clusters in Kenya (2007-2013)

Human respiratory syncytial virus (HRSV) had a positive hotspot or cluster (high-high/HH) with a p-value (p=0.004) covering counties including Kericho, Kisumu, and other adjacent counties. Besides, the positive hotspot (high-high/HH)was noted with a p-value (p=0.02) in the adjacent area of HomaBay county. However, the observed HRSV cases (High-Low/HL or Low-High/LH) with p-value (p=0.002) were an outlier cluster in the adjacent area of the Bomet county.

Human parainfluenza virus (HPIV) positive hotspot (high-high/HH) were also observed covering the Busia, Kisumu, HomaBay, and adjacent counties with a p-value range from (p=0.002) to (p=0.04). The outlier clusters (High-Low/HL or Low-High/LH) of HPIV cases were recorded in the adjacent region with p-value varies from (p=0.002) to (p=0.03).

Human adenovirus (HAdV) positive hotspots (high-high/HH) with a p-value range from (p=0.002) to (p=0.03) were located in the same area of western regions and had outlier clusters (High-Low/HL or Low-High/LH) with (p=0.03) in the neighboring areas.

**FIGURE 6:**
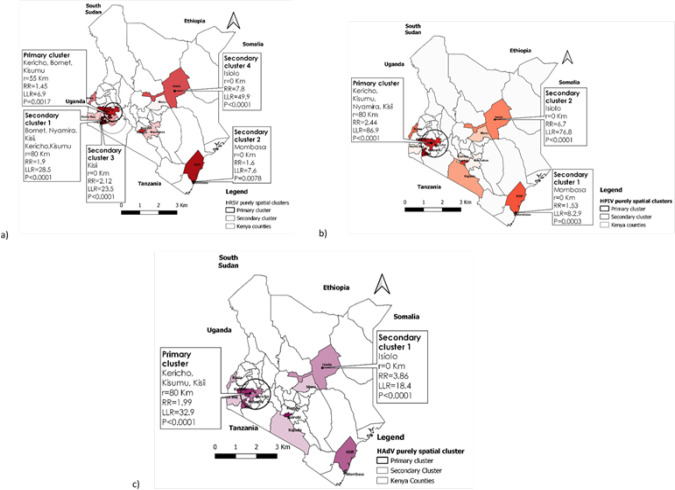
Purely spatial clustering of (a) Human respiratory syncytial virus (HRSV); (b) Human parainfluenza (HPIV); and (c) Human adenoviruses (HAdV) cases in Kenya (2007-2013)

The results of the purely spatial scan statistic revealed statistically significant (P< 0.05) primary and secondary spatial clusters of HRSV, HPIV, and HAdVs occurrence (Figure 6).

HRSV cases were concentrated in a 55-kilometer radius in the western region, encompassing three major counties. Kericho, Bomet, and Kisumu counties were included, as well as an overlapping secondary cluster with an 80-kilometer radius that included Nyamira and Kisii counties. Other secondary clusters were less likely and had a radius of less than a kilometre.

Human parainfluenza virus (HPIV) primary cluster was discovered over an 80-kilometer radius in the western region. This cluster included Kericho, Kisumu, Nyamira, and Kisii counties. Secondary clusters occurred in various regions, including the coastal area (Mombasa County) and the north eastern region (Isiolo County), with a radius of less than a Km. These secondary clusters were significantly smaller size compared to the primary cluster observed in western region.

Human adenovirus (HAdV) cases were concentrated in an 80-kilometer radius that included Kericho, Kisumu, and Kisii counties. In Isiolo County, a secondary cluster with a radius of less than one kilometre was discovered.

**FIGURE 7:**
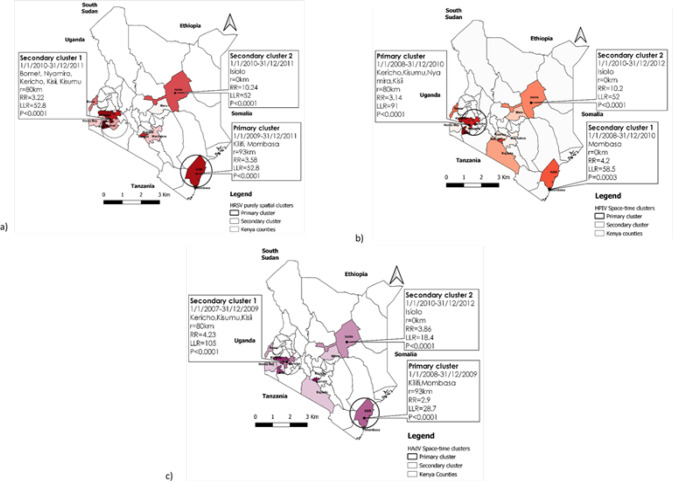
Spatiotemporal clustering of (a) Human respiratory syncytial virus (RSV); (b) Human parainfluenza (PIV); and (c) Human adenoviruses (AdV) cases in Kenya (2007-2013)

The results of spatiotemporal cluster analysis indicated different characteristics in the spatial and temporal distribution of HRSV, HPIV, and HAdV cases in Kenya (Figure 7).

From 2009 to 2011, the HRSV primary cluster had cases concentrated in a 93-kilometer radius at the coastal region, and it covered the main counties of Mombasa and Kilifi. A secondary cluster with an 80-kilometer radius covering Bomet, Nyamira, Kisii, Kericho, and Kisumu County was discovered in the western region.

This secondary cluster occurred from 2010 to 2011. HPIV primary cluster occurred from 2008 to 2010 with cases identified in Kericho, Kisumu, Nyamira, and Kisii County. This cluster had an 80-kilometer radius, while two secondary clusters in Mombasa and Isiolo counties had radiuses of less than a kilometre.

The primary cluster of HAdVs occurred within a 93-kilometer radius of the coastal region, including Kilifi and Mombasa counties, with cases identified from 2008 to 2009. From 2007 to 2009, the western region experienced a large secondary cluster of HAdV. This secondary cluster had an 80-kilometer radius and covered Kericho, Kisumu, and Kisii counties. Another secondary cluster of HAdV with a radius of less than a kilometre was recorded in Isiolo County between 2010 and 2012.

## DISCUSSION

The analysis of spatial and temporal patterns of HRSV, HPIV, and HAdV occurrence in this study provided characteristics of the influenza-like illness (ILI) distribution in Kenya from 2007 to 2013. Previous study using ILI surveillance data from the same time period reported influenza viruses causing respiratory infections. Besides that, it indicated the occurrence of other respiratory viruses in Kenya.^46^ The three respiratory viruses were found at every surveillance site that participated in the influenza-like illness (ILI) surveillance programme. The surveillance sites represented Kenya's geographical regions, including Western-Nyanza, Rift Valley, Central, Eastern, and Coastal regions.

Human respiratory syncytial virus (HRSV) occurred in 13 counties, with a high number of cases in Kisii County. The Western region was identified as the major hotspot for HRSV using Kernel density estimates (KDE), which was confirmed by local spatial autocorrelation.

Following the statistical scan, the primary cluster in this region was purely spatial. Human respiratory syncytial virus (HRSV) clustered in the Coastal region from 2009 to 2011, according to the change in time. Not surprisingly, an HRSV hotspot was also observed in the coastal region by KDE. According to a study conducted by Nyiro et al. 2018, HRSV has been identified as one of the most common pathogens causing acute respiratory tract infections in outpatients in Kenya's coastal region.^47^

Besides the purely spatial cluster in the Western region, HRSV had primary and secondary clusters over space and time for both Western and Coastal regions respectively. The HRSV clusters could be attributed to regional population characteristics, social and climate factors. Several factors, including climate parameters, have been linked to HRSV infections in previous studies.^48,49^ Rainfall and warm temperatures, which characterize the climate of the Western and Coastal regions, were the climatic conditions most commonly associated with HRSV cases.^50^

Human parainfluenza virus (HPIV) was found in 14 counties, with Kisii County having the highest number of cases. Kernel Density Estimate (KDE)indicated a major HPIV hotspot in the Western region. Other HPIV hotspots, however, were identified in the coastal and central regions. The local spatial autocorrelation indicated that the Western HPIV hotspot was statistically significant. This was confirmed with the purely spatial scan statistics. The space-time analysis revealed the occurrence of the HPIV cluster in the Western region from 2008 to 2010. Unlike HRSV infections, HPIV infections had an erratic distribution with no clear seasonality in Kenya.^50^ Furthermore, no significant climate parameters were associated with HPIV infections, as observed in other studies published elsewhere.^51^ HPIV clusters could thus be attributed to factors other than climatic parameters.

The occurrence of HAdV was documented in 11 counties, with Nairobi County having the highest number of cases. Despite the fact that the number of HAdV cases in Nairobi was high, the major hotspot was identified in the Western region, where several counties had a larger number of HAdVs. Other hotspots were found in the coastal and central regions. The major HAdV hotspot had a significant spatial autocorrelation with the Western region. This agreed with the HAdV primary cluster observed in the same region by the purely spatial scan statistics. However, space-time analysis indicated the HAdV primary cluster occurred in the Coastal region from 2008 to 2009. Human adenovirus (HAdV) cluster in the Western region, on the other hand, was secondary and occurred from 2007 to 2009. In Kenya, there were no seasonality patterns or climate parameters associated with HAdV infections. Only warm temperatures were suggested to have a positive influence on these infections.^50^ However, other factors such as population demographics, health, and socioeconomic determinants have been linked to HAdV infections in the literature^52–54^ A purely temporal analysis has previously been performed based on the same period of 2007 to 2013^50^; this study expands and augments that analysis.

A major shortfall in this study may be due to the use of annual county population projections which are based on the inter-census data; these may not accurately count the actual population per county.^55^ Also, not all county had an ILI surveillance site. Another limitation of this study could be the use of retrospective data that cannot be extrapolated to the present. The study outputs also could not be extrapolated beyond the regions without surveillance sites. Although these data-related limitations may have an impact on the study's findings, scan statistics are a well-established method for detecting disease clusters in the spatial and temporal model.^45,56–58^ They are used to analyse retrospective and prospective routine data from disease surveillance programs, and the outputs can provide insights into identifying space-time clusters, which inform intervention planning and local and national public health policies.

## CONCLUSION

Following the results of the study, HRSV, HPIV, and HAdV hotspots (clusters) occurred in Kenya's Western and Coastal regions between 2007 and 2013. Throughout the study period, the Western region appeared to be more prone to the occurrence of all three respiratory viruses. Furthermore, epidemiological studies should investigate the factors that influence the occurrence of clusters in these areas. Besides that, strategic mitigation should target those areas in order to prevent future clusters of HRSV, HPIV, and HAdV infections which could lead to epidemics.

## References

[1] Gaunt ER, Harvala H, McIntyre C, Templeton KE, Simmonds P. Disease burden of the most commonly detected respiratory viruses in hospitalized patients calculated using the disability adjusted life year (DALY) model. Journal of Clinical Virology. 2011;52(3):215–221. doi: 10.1016/j.jcv.2011.07.017 21880543 PMC7108352

[2] Shi T, McAllister DA, O'Brien KL, et al. Global, regional, and national disease burden estimates of acute lower respiratory infections due to respiratory syncytial virus in young children in 2015: a systematic review and modelling study. Lancet (London, England). 2017;390(10098):946. doi: 10.1016/S0140-6736(17)30938-8 28689664 PMC5592248

[3] Chiu SS, Chan KH, Chen H, et al. Virologically confirmed population-based burden of hospitalization caused by respiratory syncytial virus, adenovirus, and parainfluenza viruses in children in Hong Kong. Pediatr Infect Dis J. 2010;29(12):1088–1092. doi: 10.1097/INF.0b013e3181e9de24 20622713

[4] Tang JW, Lam TT, Zaraket H, et al. Global epidemiology of non-influenza RNA respiratory viruses: data gaps and a growing need for surveillance. The Lancet Infectious Diseases. 2017;17(10):e320-e326. doi: 10.1016/S1473-3099(17)30238-4 28457597 PMC7164797

[5] Kutter JS, Spronken MI, Fraaij PL, Fouchier RA, Herfst S. Transmission routes of respiratory viruses among humans. Current Opinion in Virology. 2018;28:142-151. doi: 10.1016/j.coviro.2018.01.001 29452994 PMC7102683

[6] Wang X. Global Burden of Acute Lower Respiratory Infection (ALRI) Associated with Influenza Virus, Human Metapneumovirus, and Human Parainfluenza Virus among Children under Five Years. Ph.D. University of Edinburgh; 2020. doi: 10.7488/era/405

[7] Lafolie J, Mirand A, Salmona M, et al. Severe Pneumonia Associated with Adenovirus Type 55 Infection, France, 2014. Emerg Infect Dis. 2016;22(11):2012–2014. doi: 10.3201/eid2211.160728 27767916 PMC5088017

[8] Sun B, He H, Wang Z, et al. Emergent severe acute respiratory distress syndrome caused by adenovirus type 55 in immunocompetent adults in 2013: a prospective observational study. . 2014;18(4):456. doi: 10.1186/s13054-014-0456-6 PMC4243941 25112957

[9] Janet S, Broad J, Snape MD. Respiratory syncytial virus seasonality and its implications on prevention strategies. Hum Vaccin Immunother. 2018;14(1):234–244. doi: 10.1080/21645515.2017.1403707 29194014 PMC5791579

[10] Noyola DE, Mandeville PB. Effect of climatological factors on respiratory syncytial virus epidemics. Epidemiology & Infection. 2008;136(10):1328–1332. doi: 10.1017/S0950268807000143 18177520 PMC2870732

[11] Kristensen K, Stensballe LG, Bjerre J, et al. Risk factors for respiratory syncytial virus hospitalisation in children with heart disease. Arch Dis Child. 2009;94(10):785–789. doi: 10.1136/adc.2008.143057 19541682

[12] Park SY, Kim T, Jang YR, et al. Factors predicting life-threatening infections with respiratory syncytial virus in adult patients. Infectious Diseases. 2017;49(5):333–340. doi: 10.1080/23744235.2016.1260769 27911143

[13] Kenmoe S, Bigna JJ, Well EA, et al. Prevalence of human respiratory syncytial virus infection in people with acute respiratory tract infections in Africa: A systematic review and meta-analysis. Influenza Other Respir Viruses. 2018;12(6):793–803. doi: 10.1111/irv.12584 29908103 PMC6185896

[14] Lekana-Douki SE, Nkoghe D, Drosten C, Ngoungou EB, Drexler JF, Leroy EM. Viral etiology and seasonality of influenza-like illness in Gabon, March 2010 to June 2011. BMC Infect Dis. 2014;14:373. doi: 10.1186/1471-2334-14-373 25000832 PMC4107952

[15] Dia N, Sarr FD, Thiam D, et al. Influenza-Like Illnesses in Senegal: Not Only Focus on Influenza Viruses. PLOS ONE. 2014;9(3):e93227. doi: 10.1371/journal.pone.0093227 24675982 PMC3968133

[16] Ghani ASA, Morrow BM, Hardie DR, Argent AC. An investigation into the prevalence and outcome of patients admitted to a pediatric intensive care unit with viral respiratory tract infections in Cape Town, South Africa. Pediatric Critical Care Medicine. 2012;13(5):e275. doi: 10.1097/PCC.0b013e3182417848 22596071

[17] Sanou AM, Cissé A, Millogo T, et al. Review Article Systematic Review of Articles on Etiologies of Acute Respiratory Infections in Children Aged Less Than Five Years in Sub-Saharan Africa, 2000-2015. Published online 2016:16.

[18] Obando-Pacheco P, Justicia-Grande AJ, Rivero-Calle I, et al. Respiratory Syncytial Virus Seasonality: A Global Overview. J Infect Dis. 2018;217(9):1356–1364. doi: 10.1093/infdis/jiy056 29390105

[19] Gamba-Sanchez N, Rodriguez-Martinez CE, Sossa-Briceño MP. Epidemic activity of respiratory syncytial virus is related to temperature and rainfall in equatorial tropical countries. Epidemiol Infect. 2016;144(10):2057–2063. doi: 10.1017/S0950268816000273 26888544 PMC9150586

[20] Zhao H, Harris RJ, Ellis J, Donati M, Pebody RG. Epidemiology of parainfluenza infection in England and Wales, 1998-2013: any evidence of change? Epidemiol Infect. 2017;145(6):1210-1220. doi: 10.1017/S095026881600323X 28095926 PMC9507836

[21] Dela Cruz CS, Pasnick S, Gross JE, et al. Adenovirus Infection and Outbreaks: What You Need to Know. Am J Respir Crit Care Med. ;199(7):P13-P14. doi: 10.1164/rccm.1997P13 30932693

[22] Choi M. Book Review: Spatial Analysis in Epidemiology. Healthc Inform Res. 2013;19(2):148–149. doi: 10.4258/hir.2013.19.2.148

[23] Kulldorff M, Hjalmars U. The Knox method and other tests for space-time interaction. Biometrics. 1999;55(2):544–552. doi: 10.1111/j.0006-341x.1999.00544.x 11318212

[24] Patil GP, Taillie C. Geographic and Network Surveillance via Scan Statistics for Critical Area Detection. Statist Sci. 2003;18(4):457–465. doi: 10.1214/ss/1081443229

[25] Where is Kenya in the World? Accessed April 22, 2021. https://worldpopulationreview.com/country-locations/where-is-kenya

[26] Kenya - The World Factbook. Accessed February 1, 2021. https://www.cia.gov/the-world-factbook/countries/kenya/

[27] Odhiambo EA. Geographic Classification and Geo-coding in Kenya. :24.

[28] The Independent Electoral and Boundaries Commission. The Revised Preliminary Report of the Proposed Boundaries of Constituencies and Wards.; 2012:9-12. https://www.iebc.or.ke/uploads/resources/WHXao7x83D.pdf

[29] Ayugi B, Wen W, Chepkemoi D. Analysis of Spatial and Temporal Patterns of Rainfall Variations over Kenya. Environmental Earth Sciences. 2016;6:69-83.

[30] Wakachala F, Shilenje ZW, Nguyo J, et al. Statistical Patterns of Rainfall Variability in the Great Rift Valley of Kenya. Journal of Environmental and Agricultural Sciences 2313-8629. 2015;5:17-26.

[31] Marigi S, Njogu A, Githungo W. Trends of Extreme Temperature and Rainfall Indices for Arid and Semi-Arid Lands of South Eastern Kenya. Journal of Geoscience and Environment Protection. 2016;04:158-171. doi: 10.4236/gep.2016.412012

[32] Recha C, Shisanya C, Traore P, Makokha G, Lodoun T, A. S. Determination of seasonal rainfall variability, onset and cessation in semi-arid Tharaka district, Kenya. Theoretical and Applied Climatology. ;108. doi: 10.1007/s00704-011-0544-3

[33] Mutua M. Annual and Seasonal Rainfall Variability for the Kenyan Highlands from 1900-2012. Published online September 11, 2020.

[34] Makokha G, Shisanya C. Trends in Mean Annual Minimum and Maximum Near Surface Temperature in Nairobi City, Kenya. Advances in Meteorology. 2010;2010. doi: 10.1155/2010/676041

[35] Central Bureau of Statistics-Minstry of Finance and Planning. Analytical Report on Population Projections VII: Kenya 1999 Population and Housing Census.; 2002:32.

[36] Kenya National Bureau of Statistics. Statistical Abstract 2007.; 2007:.

[37] Analytical Report on Population Projection 2010-2030. https://www.knbs.or.ke/?page_id=3142

[38] Kenya - Subnational Administrative Boundaries Humanitarian Data Exchange. Accessed March 2, 2021. https://data.humdata.org/dataset/ken-administrative-boundaries

[39] Guidoum AC. Kernel Estimator and Bandwidth Selection for Density and its Derivatives. :22.

[40] Oxoli D, Prestifilippo G, Bertocchi D, Zurbarán M. Enabling spatial autocorrelation mapping in QGIS: The hotspot analysis Plugin. Geoingegneria Ambientale e Mineraria. 2017;151:45-50.

[41] Anselin L. Local Indicators of Spatial Association—LISA. Geographical Analysis. 1995;27(2):93–115. doi: 10.1111/j.1538-4632.1995.tb00338.x

[42] Robertson C, Nelson TA, MacNab YC, Lawson AB. Review of methods for space–time disease surveillance. Spat Spatiotemporal Epidemiol. 2010;1(2):105–116. doi: 10.1016/j.sste.2009.12.001 22749467 PMC7185413

[43] Oxoli D, Molinari M, Brovelli M. Hotspot Analysis, an open source GIS tool for exploratory spatial data analysis: application to the study of soil consumption in Italy. Rendiconti Online della Societ Geologica Italiana. 2018;46:82-87. doi: 10.3301/ROL.2018.56

[44] Vilinová K. Spatial Autocorrelation of Breast and Prostate Cancer in Slovakia. Int J Environ Res Public Health. 2020;17(12). doi: 10.3390/ijerph17124440 PMC7344400 32575748

[45] Kulldorff M. A spatial scan statistic. Communications in Statistics - Theory and Methods. 1997;26(6):1481–1496. doi: 10.1080/03610929708831995

[46] Umuhoza T, Bulimo WD, Oyugi J, Schnabel D, Mancuso JD. Prevalence and factors influencing the distribution of influenza viruses in Kenya: Seven-year hospital-based surveillance of influenza-like illness (2007–2013). PLOS ONE. 2020;15(8):e0237857. doi: 10.1371/journal.pone.0237857 32822390 PMC7446924

[47] Nyiro JU, Munywoki P, Kamau E, et al. Surveillance of respiratory viruses in the outpatient setting in rural coastal Kenya: baseline epidemiological observations. Wellcome Open Res. 2018;3. doi: 10.12688/wellcomeopenres.14662.1 30175247 PMC6081997

[48] Weber A, Weber M, Milligan P. Modeling epidemics caused by respiratory syncytial virus (RSV). Math Biosci. 2001;172(2):95–113. doi: 10.1016/s0025-5564(01)00066-9 11520501

[49] Murray EL, Klein M, Brondi L, et al. Rainfall, household crowding, and acute respiratory infections in the tropics. Epidemiology & Infection. 2012;140(1):78–86. doi: 10.1017/S0950268811000252 21371367

[50] Umuhoza T, Oyugi J, Mancuso JD, Ahmed A, Bulimo WD. Morbidity burden, seasonality and factors associated with the human respiratory syncytial virus, human parainfluenza virus, and human adenovirus infections in Kenya. IJID Regions. 2021;1:72-78. doi: 10.1016/j.ijregi.2021.10.001 35757823 PMC9216343

[51] Li Y, Reeves RM, Wang X, et al. Global patterns in monthly activity of influenza virus, respiratory syncytial virus, parainfluenza virus, and metapneumovirus: a systematic analysis. Lancet Glob Health. 2019;7(8):e1031-e1045. doi: 10.1016/S2214-109X(19)30264-5 31303294

[52] Pang J, Jin J, Loh JP, et al. Risk factors for febrile respiratory illness and mono-viral infections in a semi-closed military environment: a case-control study. BMC Infectious Diseases. 2015;15:288. doi: 10.1186/s12879-015-1024-7 26208494 PMC4514976

[53] Walls T, Shankar AG, Shingadia D. Adenovirus: an increasingly important pathogen in paediatric bone marrow transplant patients. Lancet Infect Dis. 2003;3(2):79–86. 12560192 10.1016/s1473-3099(03)00515-2

[54] Bautista-Gogel J, Madsen CM, Lu X, et al. Outbreak of Respiratory Illness Associated With Human Adenovirus Type 7 Among Persons Attending Officer Candidates School, Quantico, Virginia, 2017. The Journal of Infectious Diseases. 2020;221(5):697–700. doi: 10.1093/infdis/jiz060 30783668

[55] Trizer M. 2019 Kenya Population and Housing Census Results. Kenya National Bureau of Statistics. Published November 4, 2019. Accessed October 8, 2020. https://www.knbs.or.ke/?p=5621

[56] Tango T, Takahashi K. A flexible spatial scan statistic with a restricted likelihood ratio for detecting disease clusters. Stat Med. 2012;31(30):4207–4218. doi: 10.1002/sim.5478 22807146

[57] Rao H, Shi X, Zhang X. Using the Kulldorff's scan statistical analysis to detect spatio-temporal clusters of tuberculosis in Qinghai Province, China, 2009–2016. BMC Infect Dis. 2017;17. doi: 10.1186/s12879-017-2643-y 28826399 PMC5563899

[58] Gwitira I, Mukonoweshuro M, Mapako G, Shekede MD, Chirenda J, Mberikunashe J. . Infectious Diseases of Poverty. ;9():146. doi: 10.1186/s40249-020-00764-6 PMC7584089 33092651

